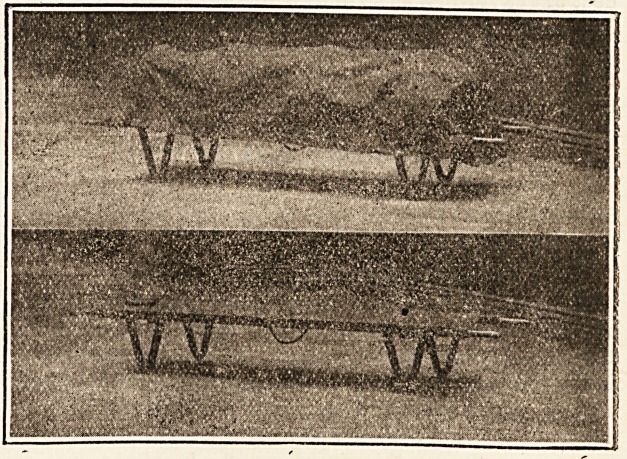# New Appliances & Things Medical

**Published:** 1911-04-08

**Authors:** 


### NEW APPLIANCES & THINGS MEDICAL.

### A NEW CARRIAGE-STRETCHER.

Mr. Allen Maldrett, Superintendent and Secretary of the Royal Southern Hospital, writes to us as follows: "Herewith I send you photographs of a stretcher and carriage recently designed by myself and made for this hospital by Messrs. W. Harrison and Co., of No. 73 Bold Street, Liverpool. Its construction was dictated by a want, common to most hospitals, of a carriage which would accommodate a light stretcher for various uses in hospitals, and also the stretchers belonging to the police and other ambulances. The carriage has proved of immense use in this latter connection—patients badly injured being removed directly to the wards on the stretcher conveying them to hospital. The carriage has two swivel wheels and two fixed front wheels on springs, all with ball bearings, and can be manipulated with the greatest ease. The stretcher, it will be seen, has legs with small wheels, and stands at a convenient height for use at the bedside of mortuary tables; the handles are folding, with strong hinges, reducing its length when required to go into small lifts. It is also fitted with a series of sockets, which carry hoops, supporting the canvas cover which is used for the conveyance through hospital of bodies brought in dead. I venture to think that this carriage and stretcher will interest many of your readers and supply a long-felt want in many hospitals.

## Figures and Tables

**Figure f1:**
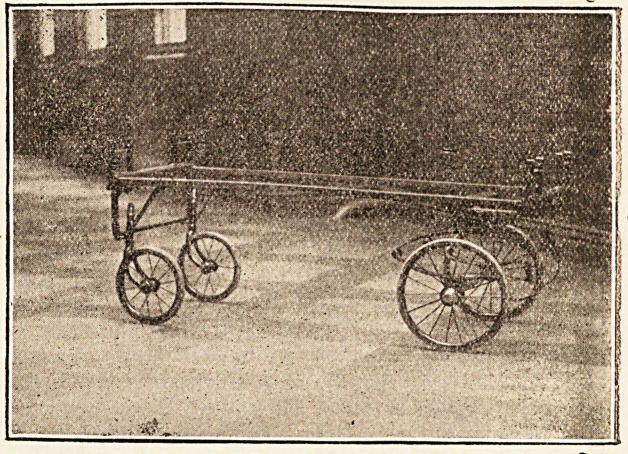


**Figure f2:**